# Supplementary material for: Genotype by environment interaction across water regimes in relation to cropping season response of quinoa (Chenopodium quinoa)
Source: PLoS One. 2024 Oct 8;19(10):e0309777. doi: 10.1371/journal.pone.0309777 (PMC11460699; doi:10.1371/journal.pone.0309777)
Supplement: Attachment — Submitted filename: Response to Reviewers.docx [file pone.0309777.s001.docx]

***August 6^th^ 2024***

**Dear** Editor *of Plos One*

**Dear Reviewers**

Firstly, let us give our gratitude for your valuable recommendations

Following your recommendations and the reviewer’s comments, we have revised the manuscript accordingly (revisions are highlighted in track change). Below are our responses to reviewer´s specific comments.

Thanks so much again for your support !

Sincerely yours,

Authors,

**Authors’ revision for comments**

**Reviewer 1:**

**Thank you very much for appreciating the value of our manuscript with minor comments**

Below are our responses to your specific comments.

***Comment #1:*** The hydroponic growth should be mentioned for experiment 1. That will help readers can link to the the term hydroponic at the end part of the abstract.

***Authors’ response for comment #1***: Thank you very much for your comment. We’ve mentioned hydroponic growth for experiment 1 in Abstract accordingly:

Waterlogging and drought treatments were artificially induced using normoxia, a combination of hypoxia-normoxia, and 10% PEG (Polyethylene glycol) under hydroponic growth conditions, respectively

***Comment #2:*** Does the G18 has the same response between two two experiments (basing on GGE plots)?
***Authors’ response for comment #2***: Thank you very much for your comment. We found that G18 is a good material, showing water stress tolerance under experiments, including field trials which will be discussed in our next paper

***Comment #3:*** Plants were treated (waterlogging, drought) immediately when seedlings were transferred to the hydroponic growth?

***Authors’ response for comment #3:*** Thank you very much for your question. This is to clarify the experimental method. We’ve checked and added information about the drought stress treatments (no need to adjust the shock due to oxygen deficiency because the oxygen reduction treatment simulates real conditions) in the Materials and Methods section accordingly:

Under drought conditions, plants were grown for 14 days with 10% w/v PEG6000. To avoid drought shock injury, PEG6000 was applied stepwise to the basic nutrient solution. This began after a period of 6 hours, increasing the concentration in 5% increments on the first day of treatment

***Comment #4:*** Line 101: English expressions, each genotype in Table 1 was grown... instead of "plants of each of the genotypes".

***Authors’ response for comment #4:*** Thank you very much for your comments. We’ve revised accordingly

***Comment #5:*** Table 1: Why select those 20 genotypes for hydroponic experiment?

***Authors’ response for comment #5:*** Thank you very much for your comments. We expected to proceed with all the materials; however, due to the volume of the experiment, hydroponic culture is complex and requires substantial labor and specific experimental conditions. Therefore, we have chosen 20 genotypes with diverse origins to use.

***Comment #6:*** The calculation of WTI and DTI should be explained clearer (line 138-140). Were they calculated by divided traits under treatments (waterlogging or drought) by under control?

***Authors’ response for comment #6:*** Thank you very much for your comments. We’ve checked, disscussed and thought that the sentence “Waterlogging tolerance index (WTI) and drought tolerance index (DTI) were calculated as the waterlogging and drought versus control ratio for each measured trait, respectively” is clear enough.

***Comment #7:*** Leaf area was measured for total leaves? or a specific leaf type?

***Authors’ response for comment #7:*** Thank you very much for your comments, leaf area was measured for total leaves

***Comment #8:*** Term should be used consistently (Table 4. Hypoxia tolerance index – HTI. This does not present in the table)

***Authors’ response for comment #8:*** Thank you very much for your comments. HTI was our mistake, we’ve changed HTI to WTI.

***Comment #8:*** What is “test environments” (line 198)?

***Authors’ response for comment #8:*** Thank you very much for your comments, we’ve revised test environments to water stress invironments

***Comment #9:*** Why PC1 and PC2 make up 100% (line 205)? Normally less than 100%

***Authors’ response for comment #9:*** Thank you very much for your comments. We have checked and explained that we ran principal components (PCs) for a single trait of genotypes under three environments so that PC1 and PC2 account for 100% of the variation. This approach is consistent with all previous research

***Comment #10:*** Line 207: not grain yield?

***Authors’ response for comment #10:*** Thank you very much for your comments. We have checked and revised accordingly.

***Comment #11:*** GGE plots did not show similar response between hydroponic and field growth (i.e., G18)?
***Authors’ response for comment #11:*** Thank you very much for your comments. We agree with you, as field growth was influenced by other environmental factors.

***Comment #12:*** Line 273-274: does not make sense since drought tolerance does not cover all abiotic stressors.

***Authors’ response for comment #12:*** Thank you very much for your comments. We have checked and revised accordingly: Although quinoa is considered a drought-tolerant crop, its growth can still be significantly reduced by water deficit stress.

**Reviewer 2:** The paper is directed on quinoa, a novel crop that shall increase its importance worldwide. It is well written; the statistics is correct and provide basic information to support discussion and conclusions. Given this, it is worth publishing. However, I suggest it be submitted to a more specialized journal, with focus on genetics and breeding. In this way it may reach the public involved in crop improvement.

**Authors’ response for comment: Thank you very much for appreciating the value of our manuscript with recommendation for** specialized journal. We will consider for our next papers.

**Journal comments:**

We've checked your submission and before we can proceed, we need you to address the following issues:

1.In your Methods section, please provide additional information regarding the permits you obtained for the work. Please ensure you have included the full name of the authority that approved the field site access and, if no permits were required, a brief statement explaining why.

**Authors’ response for comment:**

We have added the information on field site acess: . In Experiment 1, plants of each genotype in Table 1 were grown in hydroponic conditions under semi-controlled conditions in a glasshouse of the Faculty of Agronomy, Vietnam National University of Agriculture (latitude 20^o^ 60’N, longitude 105^o^ 56’W, altitude ~20 m.a.s.l.). In Experiment 2, plants of each of the 58 genotypes of quinoa (Table 1) were sown at the upland field trial site of the Faculty of Agronomy, Vietnam National University of Agriculture in the winter (i.e. month 9 to month 12) and spring (i.e. month 2 to month 6) cropping seasons.

The research site and facilities belong to the Vietnam National University of Agriculture, with staff assigned to these sites and facilities.

2.Thank you for stating the following financial disclosure:"This research was supported by a Vietnam Ireland Bilateral Education Exchange (VIBE) program grant (funded by the Irish Department of Foreign Affairs (Irish Aid) to Vietnam National University of Agriculture (Vietnam) and University of Galway (Ireland) to VLN and CS, respectively. The VIBE program grant was titled “Quinoa development for food security under climate change conditions in Vietnam”, Pillar 01-2022.04/VNUA 01."

Please state what role the funders took in the study.
If the funders had no role, please state:
 "The funders had no role in study design, data collection and analysis, decision to publish, or preparation of the manuscript."
If this statement is not correct you must amend it as needed.
Please include this amended Role of Funder statement in your cover letter;
we will change the online submission form on your behalf.

**Authors’ response for comment: we stated**  "The funders had no role in study design, data collection and analysis, decision to publish, or preparation of the manuscript."

3.Please include your full ethics statement in the ‘Methods’ section of your manuscript file. In your statement, please include the full name of the IRB or ethics committee who approved or waived your study, as well as whether or not you obtained informed written or verbal consent. If consent was waived for your study, please include this information in your statement as well.

**Authors’ response for comment: We added accordingly in “Methods”:** This research was supported by a Vietnam Ireland Bilateral Education Exchange (VIBE) program grant (funded by the Irish Department of Foreign Affairs (Irish Aid) to Vietnam National University of Agriculture (Vietnam) and University of Galway (Ireland). The VIBE program grant was titled “Quinoa development for food security under climate change conditions in Vietnam”, Pillar 01-2022.04/VNUA 01.

4."In the online submission form, you indicated that [Data are available from the Institutional Data Access for researchers who meet the criteria for access to confidential data.].

All PLOS journals now require all data underlying the findings described in their manuscript to be freely available to other researchers, either
1. In a public repository,
2. Within the manuscript itself, or
3. Uploaded as supplementary information.

This policy applies to all data except where public deposition would breach compliance with the protocol approved by your research ethics board. If your data cannot be made publicly available for ethical or legal reasons (e.g., public availability would compromise patient privacy), please explain your reasons on resubmission and your exemption request will be escalated for approval."

**Authors’ response for comment:** We’ve added accordingly

5."Please amend your list of authors on the manuscript to ensure that each author is linked to an affiliation.
We note that you have included affiliation numbers 1,2,3,4,5 and 6 however only affiliations 1,3,4,5 and 6 have authors linked to them.  Please amend affiliation 2 to link an author to it or remove if added in error.

**Authors’ response for comment:** We’ve revised accordingly
